# Histone deacetylase inhibitors provoke a tumor supportive phenotype in pancreatic cancer associated fibroblasts

**DOI:** 10.18632/oncotarget.13572

**Published:** 2016-11-24

**Authors:** Andrew H. Nguyen, Irmina A. Elliott, Nanping Wu, Cynthia Matsumura, Maria Vogelauer, Narsis Attar, Amanda Dann, Razmik Ghukasyan, Paul A. Toste, Sanjeet G. Patel, Jennifer L. Williams, Luyi Li, David W. Dawson, Caius Radu, Siavash K. Kurdistani, Timothy R. Donahue

**Affiliations:** ^1^ Department of Surgery, David Geffen School of Medicine at UCLA, Los Angeles, California, USA; ^2^ Department of Biological Chemistry, David Geffen School of Medicine at UCLA, Los Angeles, California, USA; ^3^ Department of Surgery, Harbor-UCLA Medical Center, Torrance, California, USA; ^4^ Department of Pathology and Laboratory Medicine, David Geffen School of Medicine at UCLA, Los Angeles, California, USA; ^5^ Department of Molecular and Medical Pharmacology, David Geffen School of Medicine at UCLA, Los Angeles, California, USA

**Keywords:** histone deacetylase inhibitor, SAHA, AP-1, pancreatic cancer, cancer-associated fibroblasts

## Abstract

Although histone deacetylase inhibitors (HDACi) are a promising class of anti-cancer drugs, thus far, they have been unsuccessful in early phase clinical trials for pancreatic ductal adenocarcinoma (PDAC). One potential reason for their poor efficacy is the tumor stroma, where cancer-associated fibroblasts (CAFs) are a prominent cell type and a source of resistance to cancer therapies. Here, we demonstrate that stromal fibroblasts contribute to the poor efficacy of HDACi's in PDAC. HDACi-treated fibroblasts show increased biological aggressiveness and are characterized by increased secretion of pro-inflammatory tumor-supportive cytokines and chemokines. We find that HDAC2 binds to the enhancer and promoter regions of pro-inflammatory genes specifically in CAFs and *in silico* analysis identified AP-1 to be the most frequently associated transcription factor bound in these regions. Pharmacologic inhibition of pathways upstream of AP-1 suppresses the HDACi-induced inflammatory gene expression and tumor-supportive responses in fibroblasts. Our findings demonstrate that the combination of HDACi's with chemical inhibitors of the AP-1 signaling pathway attenuate the inflammatory phenotype of fibroblasts and may improve the efficacy of HDACi in PDAC and, potentially, in other solid tumors rich in stroma.

## INTRODUCTION

Pancreatic ductal adenocarcinoma (PDAC) is associated with poor overall prognosis and resistance to both conventional and emerging therapies. Gemcitabine is the most frequently used chemotherapy for PDAC, but at best, it increases survival by just a few weeks in both early- and advanced-stage patients [1]. Over the last 10 years, there have only been three new drugs approved by the FDA for PDAC, each of which confers only modest survival improvement in advanced-stage patients. In 2005, erlotinib, an EGFR inhibitor, was approved after being shown to increase survival by an average of 10 days as compared to gemcitabine alone [2]. In 2014, a nanoparticle coated with albumin and packaged with paclitaxel (nab-paclitaxel) was also approved after demonstrating 2 months additional survival over gemcitabine [3]. Recently, liposomal irinotecan combined with fluorouracil/leucovorin has been approved for advanced-stage disease, also adding a modest 2 months additional survival compared to fluorouracil alone. As PDAC is predicted to become the second leading cause of cancer-related deaths in the United States within the next 10 years, there is an ever-pressing need to identify new therapies that are broadly effective against this particularly difficult to treat malignancy [4].

Histone deacetylase inhibitors (HDACi’s) are a chemically diverse class of anti-cancer drugs that function by inhibiting lysine deacetylases, resulting in greater acetylation of histones by histone acetyltransferases (HATs) and transcriptional de-repression of their target genes. In tumor cells (TC), HDAC inhibition predominantly induces G1-S cell cycle arrest by increasing CDKN1A/p21 expression [5, 6]. HDACi's have shown greatest promise in the treatment of hematologic malignancies. FDA approval was granted for the pan-HDACi vorinostat (suberanilohydroxamic acid/SAHA) in 2006 [7], and class I selective HDACi romidepsin in 2009 for refractory cutaneous T-cell lymphoma [8] and in 2011 for other peripheral T-cell lymphomas [9]. In 2015, panobinostat, another pan-HDACi, was approved for the treatment of multiple myeloma [10]. However, early phase clinical trials of HDACi’s, such as vorinostat/SAHA and panobinostat/LBH-589, combined with standard-of-care cytotoxic chemotherapy for solid tumors, including PDAC, did not show any therapeutic benefit [11, 12]. Although HDACi's appear to be effective against solid tumor cancer cell lines, including PDAC, in culture and *in vivo*, these preclinical results have not been reproduced in early phase clinical trials [13].

As compared to most hematologic malignancies, many solid organ tumors contain a diversity of cellular and non-cellular components in the peritumoral microenvironment, collectively referred to as the “tumor-associated stroma.” PDAC has the highest stromal volume that comprises approximately 80 percent of the tumor mass, including a cellular compartment comprised predominantly of cancer-associated fibroblasts (CAFs). In PDAC, CAFs contribute to treatment resistance by synthesizing extracellular matrix proteins that impede drug delivery, and also secrete cytokines and chemokines, which act on neighboring TCs via paracrine signaling. These secreted factors derived from PDAC CAFs increase TC proliferation, migration, invasion, and colony formation in cell culture and *in vivo* [14–16].

In this study, we hypothesized that PDAC CAFs contribute to the poor efficacy of HDACi's in PDAC and therefore evaluated the effects of HDACi's on PDAC CAFs in culture and *in vivo*. We found that PDAC CAFs treated with HDACi's paradoxically become pro-tumorigenic. SAHA transiently increased proliferation of PDAC TCs when co-cultured with CAFs, a phenotype mediated by a pro-inflammatory secretory response induced in CAFs by HDACi’s. ChIP-sequencing for HDAC2 revealed enrichment upstream of pro-inflammatory genes that are also commonly regulated by the AP-1 transcription factors. Finally, blockade of this pro-inflammatory response with JNK inhibitors markedly attenuated the tumor-promoting HDACi-induced inflammatory response in these CAFs.

## RESULTS

### HDACi's decrease PDAC tumor cell viability in isolation but not in the presence of CAFs

**Figure 1:**
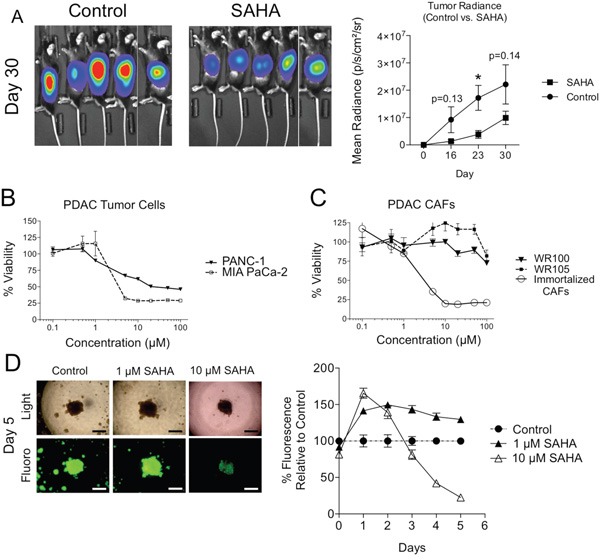
Effect of HDAC inhibition on PDAC tumor cell viability in isolation and in 3D co-culture with CAFs **A**. KPC-luc cells were implanted in the pancreata of C57BL/6 mice and mice were treated with oral gavage of SAHA 5 times weekly beginning on Day 7. Luminescence measurements were measured on days 16, 23, and 30 in SAHA treated and control mice. Error bars represent SEMs. *P < 0.05. **B**. MTT assay of PANC-1 and MIA PaCa-2 PDAC tumor cell viability at various doses of SAHA for 96h. **C**. MTT assay of WR100 and WR105 primary PDAC fibroblasts and immortalized CAFs at various doses of SAHA for 96h. **D**. Images of PANC-1-GFP/PDAC fibroblast co-culture under fluorescence and light microscopy (left) (4X; scale: 300 μm) and 5 day time course of relative fluorescence of PANC-1-GFP/PDAC CAF co-cultures with treatment of SAHA. Fluorescence is normalized to untreated control cell co-cultures.

In a syngeneic, orthotopic KrasLSL.G12D/+; p53R172H/+; PdxCretg/+ (KPC) PDAC mouse model, systemic treatment with the pan-HDACi SAHA suppressed tumor growth as compared to untreated controls (Figure 1A, Supplementary Figure 1). Analysis of untreated or treated tumor explants on day 37 after implantation revealed that they were almost entirely comprised of TCs with homogeneous morphology (Supplementary Figure 1B) and without the typical dense fibrotic stroma typical of human PDAC histology (Supplementary Figue 2). Therefore, we hypothesized that SAHA was effective in this model because of the lack of the typical stroma which comprises up to 80% of human PDAC.

We observed that immortalized PDAC cells are sensitive to HDACi in culture. MIA PaCa-2 cells were highly sensitive to SAHA with an IC50 of 4 μM (Figure 1B). PANC-1 cells were also sensitive after 96h, albeit with a higher IC50 (10 μM) (Figure 1B). In contrast to TCs, two patient-derived primary PDAC CAF lines were extremely resistant to SAHA across a wide range of doses (Figure 1C). The resistance was not unique to SAHA as CAFs were also resistant to either the Class I selective HDACi entinostat (MS-275) or the pan-HDACi panobinostat (LBH-589), both of which decreased MIA PaCa-2 viability (Supplementary Figure 3). Interestingly, hTERT immortalized PDAC CAFs were found to be highly sensitive to SAHA, which is similar to MIA PaCa-2 cancer cells with an IC50 of 4 μM (Figure 1C). For this reason, in subsequent experiments, we utilized only early passage non-immortalized patient-derived primary PDAC CAFs.

We next asked if the presence of fibroblasts affected TC sensitivity to HDACi’s. Using a 3D TC:CAF co-culture model on Matrigel, GFP-expressing PANC-1 TCs formed 3D tumor-like spheres only when cultured in the presence of CAFs (Figure 1D) where CAFs are evenly distributed among TCs (Supplementary Figure 4). PANC-1 cells cultured in the absence of fibroblasts formed only small clusters of cells rather than a larger sphere (Supplementary Figure 5). To quantify tumor cell proliferation while in TC:CAF co-culture, measurements of fluorescence from GFP expressing cancer cells was performed. Fluorescence measurements using a multimode microplate reader in this heterocellular model were validated with the Cell Titer Glo assay as an accurate measure of TC numbers (Supplementary Figure 6). Additionally, GFP-expressing TCs were treated with SAHA and the Cell Titer Glo assay was performed and we observed the addition of SAHA did not affect the linear relationship between GFP fluorescence measurements and cellular viability (Supplementary Figure 7). While PANC-1 cells cultured alone on plastic (Figure 1B) or Matrigel (Supplementary Figure 8) were sensitive to HDACi, both longer exposure and higher doses were required to suppress TC proliferation in the 3D TC:CAF co-culture model (Figure 1D). Intriguingly, PANC-1 cells in the TC:CAF model were more proliferative with SAHA treatment at early time points during continuous treatment compared to untreated controls (see days 1 and 2 in Figure 1D). Taken together, these results suggest that the HDACi-treated PDAC TCs benefit from the presence of tumor supportive CAFs.

### HDACi-treated CAFs enhance aggressiveness of neighboring tumor cells via a paracrine mechanism

**Figure 2:**
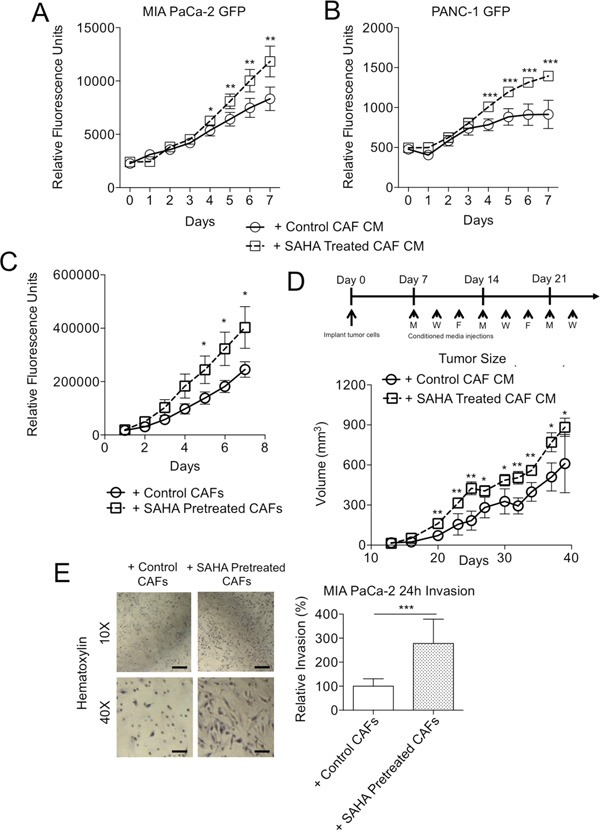
HDACi-treated fibroblasts enhance tumor cell malignant phenotypes Fluorescence measurements of **A**. MIA PaCa-2-GFP and **B**. PANC-1-GFP cells cultured with conditioned media (without drug) from CAFs pre-treated with or without SAHA for 7 days. **C**. Fluorescence measurements of PANC-1-GFP 3D co-cultured (without drug) for 7 days with CAFs pre-treated +/− SAHA. **D**. Schematic of *in vivo* experimental design (above) and tumor volume (below) of subcutaneous flank MIA PaCa-2 tumors of 8 tumors per group injected thrice weekly with conditioned media from cultured CAFs +/− SAHA. *P < 0.05, **P < 0.01. **E**. Light microscopy images (left) (10X; scale: 100 μm, 40X; scale 10 μm) and relative invasion (right) of MIA PaCa-2 invasion with control or 10 μM SAHA pre-treated PDAC CAFs after 24h in a modified Boyden chamber. *P < 0.05, **P < 0.01, ***P < 0.001.

We next determined how HDACi-treated CAFs modulate various TC phenotypes through a series of cell culture and *in vivo* models. First, both MIA PaCa-2 and PANC-1 TCs were more proliferative when cultured in conditioned media (CM) from CAFs pre-treated with HDACi than CM from untreated controls as measured by plate fluorescence (Figure 2A–2B). Similarly, PANC-1 TCs grew faster in the TC:CAF Matrigel model with CAFs that had been pre-treated with SAHA than untreated controls (Figure 2C). Finally, we also examined if HDACi-treatment of the same patient-derived CAFs could also enhance tumor growth *in vivo*. MIA PaCa-2 cells were implanted in bilateral subcutaneous flanks of NOD/SCID/IL2γ mice and tumors were supplemented thrice weekly with CM (without drug) from cultured CAFs pre-treated +/− SAHA (Figure 2D). Consistent with the cell culture results, tumor growth was accelerated, particularly at earlier time points, in mice injected with SAHA-treated CAF CM.

In addition to enhancing cell proliferation, pretreatment of CAFs with HDACi increased the invasion of co-cultured TCs. CAFs were included in this assay, as their presence has been shown to be important for the process of TC invasion [17]. We observed significantly greater invasion of MIA PaCa-2 cells when cultured in the presence CAFs that had been pre-treated with SAHA as compared to untreated control CAFs (Figure 2E). Taken together, these results suggest that HDACi treatment causes CAFs to become more supportive of tumor growth and aggressiveness in cell culture and *in vivo* through a paracrine mechanism.

### SAHA treatment increases expression of tumor-supportive, pro-inflammatory mediators in CAFs

**Figure 3:**
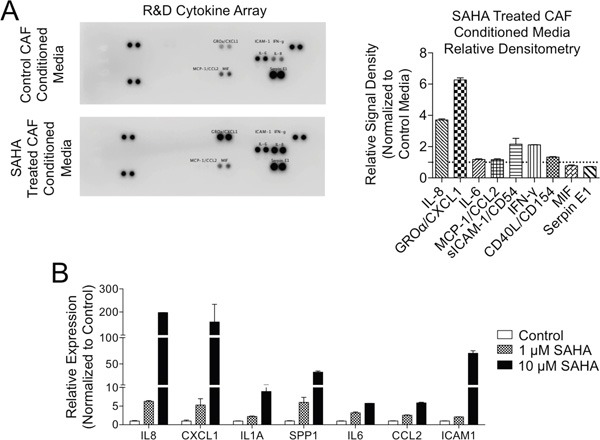
HDACi treatment increases secretion of tumor supportive pro-inflammatory mediators in PDAC CAFs **A**. Secretion of pro-inflammatory cytokines was assessed after 24h in control and 10 μM SAHA treated CAFs by cytokine array (left) with relative densitometry measurements (right). **B**. Gene expression of inflammatory mediators was determined by qRT-PCR in PDAC CAFs treated for 24h with SAHA.

A key mechanism by which CAFs modify the behavior of neighboring TCs is via release of pro-inflammatory factors into the tumor microenvironment [15, 16, 18, 19]. Given our findings with CM from HDACi-treated CAFs that indicated a paracrine mechanism of action, we explored whether pro-inflammatory mediators were increased following HDACi-treatment of CAFs. We initially compared the composition of CM from PDAC CAFs treated with SAHA vs. untreated controls using a membrane-based antibody cytokine array and found HDACi treatment was associated with increased production of pro-inflammatory mediators CXCL1 and IL-8 (Figure 3A), which are known to enhance TC malignant phenotypes [20]. These findings were further validated in a panel of pro-inflammatory genes by quantitative RT-PCR which demonstrated SAHA treatment caused a dose-dependent increase in the expression of this panel of inflammatory genes linked to the tumor-supportive senescence associated secretory phenotype (SASP) [16, 21], including IL8, CXCL1, IL1A, SPP1, IL6, CCL2, and ICAM1 (Figure 3B). To determine if the enhanced expression of pro-inflammatory mediators was unique to the pan-HDACi SAHA, we next treated primary PDAC CAFs with class I selective HDACi entinostat/MS-275 or the highly potent pan-HDACi panobinostat/LBH-589 (Supplementary Figure 9), each of which also increased the expression of the same panel of genes.

### HDACi effects on gene expression are cell-type specific

**Figure 4:**
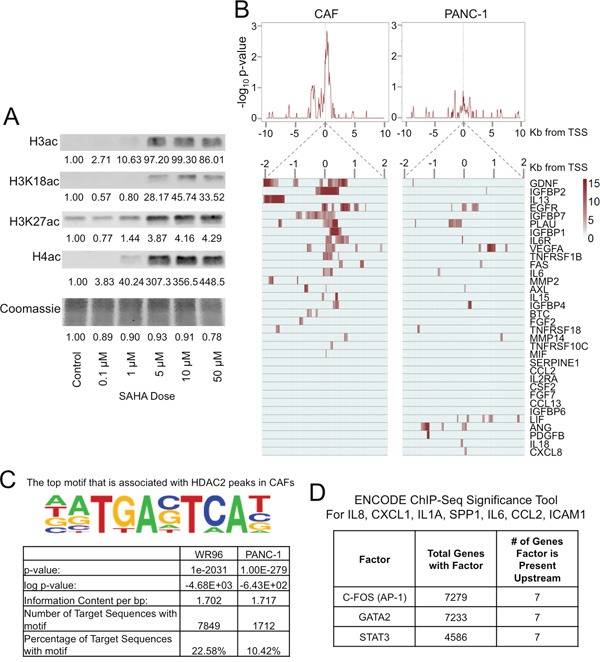
HDAC inhibition causes histone hyperacetylation in CAFs **A**. Near-infrared western blots of histone extracts of PDAC CAFs treated at various doses of SAHA for 24h. Quantitative near-infrared fluorescent western blots were performed using the LI-COR Odyssey Fc imaging system with densitometry measurements were performed relative to untreated control cell blots are reported. **B**. HDAC2 ChIP-Seq was performed in primary PDAC CAFs and PANC-1 TCs. Average profile Distribution of HDAC2 binding ± 10 Kb from the transcription start site (TSS) of pro-inflammatory genes (top) and heat map of HDAC2 distribution ± 2.5 Kb from the TSS (below) of individual pro-inflammatory genes using significant peaks (P = 10^4^ over three 50 bp windows). **C**. Transcription factor binding motif analysis using HOMER (Hypergeometric Optimization of Motif EnRichment). The top motif identified with HDAC2 peaks in CAFs was found to be associated with AP-1 transcription factor as displayed (P = 1.0 × 10^−2031^). This motif was present in 22% of HDAC2 peak sequences in fibroblasts. **D**. Factors bound to chromatin common among 7 pro-inflammatory genes within 5,000 base pairs of the transcription start site of these genes were identified using the ENCODE ChIP-Seq Significance Tool (q < 0.05). STAT3, C-FOS (a component of the AP-1 heterodimer), and GATA2 were found to commonly bind in gene regulatory regions in the vicinity of the TSS of all 7 genes.

We next determined the mechanism by which HDACi's increase the expression of pro-inflammatory genes in PDAC CAFs. Histone hyperacetylation through HDAC inhibition results in a permissive transcriptional landscape and the expression of HDAC-regulated genes [22]. In PDAC CAFs, we found that increasing doses of HDACi resulted in increased global histone acetylation (Figure 4A) with the threshold for histone hyperacetylation occurring in the range of 5–10 μM SAHA; therefore, 10 μM HDACi was used for subsequent experiments.

To determine whether HDACs may directly suppress the expression of pro-inflammatory genes in CAFs, we performed ChIP-Seq for HDAC2 in primary PDAC CAFs and the PANC-1 TC line. HDAC2 is known to have a role in cell cycle dysregulation in cancer [23, 24]; it is highly expressed in PDAC and associated with resistance to therapy and apoptosis [25, 26]. However, the role of HDAC2 in CAFs is not well defined. In CAFs, we found that HDAC2 binding is enriched in the regulatory regions primarily within 2 Kb of the transcription start site of a large group of pro-inflammatory genes, which was not present in PANC-1 cells (Figure 4B).

In contrast, using GREAT gene ontology analysis [27], HDAC2 binding in PANC-1 TCs occurs in genes predominantly involved in growth factor signaling and cell cycle regulation (Supplementary Figure 10). Induction of cell cycle-related CDKN1A/p21 expression by HDACi's in TCs is believed to be a key mechanism for the resultant cell cycle arrest [5, 22, 28].

These distinct HDAC2 binding patterns in CAFs and TCs may explain differential effects HDACi's have on these two cell types. We next used the HOMER Motif Analysis to identify the enrichment of transcription factor motifs in ChIP-Seq data [29], and found that HDAC2 binds to motifs associated with the AP-1 transcription both PANC-1 TCs and in CAFs although AP-1 motifs were more frequent in CAFs (Figure 4C). These findings were similarly replicated in a search using the Cistrome SeqPos Motif Analysis [30], which identified the c-JUN/AP-1 related binding motifs to be present in the vicinity of HDAC2 binding in CAFs (Supplementary Figure 11).

We further broadly queried common transcription factors that regulate a panel of pro-inflammatory genes upregulated after HDACi treatment in CAFs using the ENCODE ChIP-Seq Significance Tool [31]. This tool identifies transcription factors that commonly regulate sets of genes by searching publically available ChIP-Seq data sets from the ENCODE Project. We searched for DNA binding factors present within 5,000 base pairs upstream and downstream of the transcription start site of the seven SASP pro-inflammatory genes consistently increased with HDACi treatment in our studies: IL8, CXCL1, IL1A, SPP1, IL6, CCL2, and ICAM1 (Figure 4D). This tool identified STAT3, c-FOS (a component of AP-1), and GATA2 (q<0.05) to be in regulatory regions of these genes. As AP-1 is a well known regulator of inflammation [32] and our HDAC2 ChIP-Seq studies suggest the greatest association of HDAC2 to AP-1 transcription factor binding motifs, we chose to further investigate blockade of the upstream pathways of AP-1 signaling to inhibit the CAF-mediated inflammatory response to HDACi’s.

### JNK1 inhibition potently suppresses pro-inflammatory, tumor-supportive changes in HDACi-treated CAFs

A key step in forming the active AP-1 transcription factor includes the phosphorylation of c-JUN by Janus N-terminal Kinases (JNKs) to allow heterodimerization of the c-Fos/c-JUN complex. JNK has been implicated as a key regulator of a feed-forward loop of inflammatory signaling, amplifying the production of pro-inflammatory cytokines including IL-8 [33]. JNK inhibition has additionally been shown to effectively inhibit PDAC growth in culture and *in vivo*, using transgenic [34] and human tumor xenograft models [35]. Our previous results have also shown that inhibition of stress response kinases, including JNK, attenuates the inflammatory response in CAFs to DNA damaging chemotherapies [16]. Thus, taken together with our in silico results with HDAC2 ChIP-Seq, we focused the remainder of our studies on JNK inhibition as a potentially effective co-therapy with HDACi.

**Figure 5:**
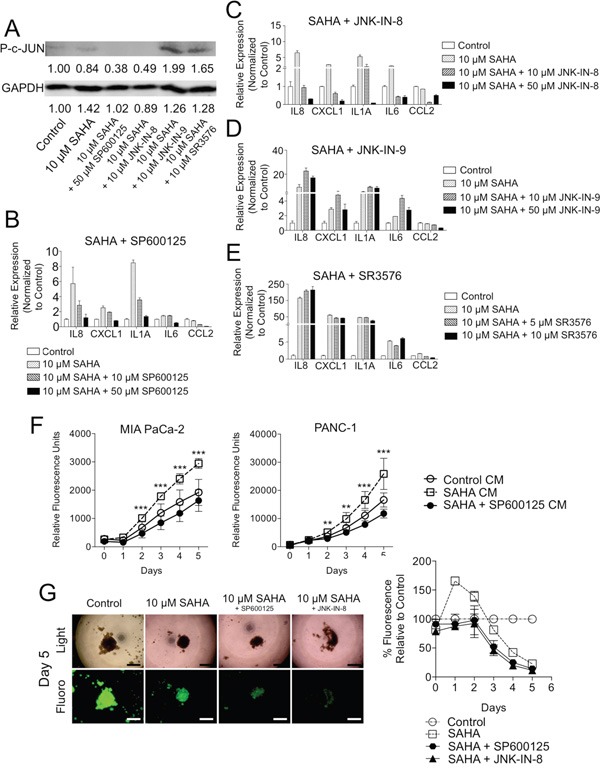
Chemical inhibition of JNK suppresses tumor supportive effects of HDACi in CAFs **A**. PDAC CAFs were treated with SAHA +/− various JNK inhibitors and western blots for P-c-JUN and GAPDH was performed of cell lysates with measurements of relative densitometry reported below blot signals. qRT-PCR was performed of PDAC CAFs treated with SAHA and various doses of **B**. SP600125, **C**. JNK-IN-8, **D**. JNK-IN-9, or **E**. SR3576. **F**. Fluorescence measurements of GFP-expressing MIA PaCa-2 (left) and PANC-1 (right) cells grown in conditioned media (without drug) from i) naïve CAFs or ii-iii) CAFs pretreated with 10 μM SAHA+/− 50 μM SP600125. T-tests were performed to compare 10 μM SAHA CM and 10 μM SAHA + 50 μM SP600125 CM groups (**P < 0.01, ***P < 0.001). **G**. PANC-1-GFP / CAF 3D co-cultures were treated with SAHA +/− JNK inhibitors: 50 μM SP600125 or 10 μM JNK-IN-8 and fluorescence measurements were performed daily (4X; scale: 300 μm).

The JNK family of kinases consists of three different isoforms (JNK 1, 2, or 3), which regulate a diverse set of pathways with variable activity based on cell-specific context [36]. We examined a panel of non-specific and isoform-specific JNK inhibitors. We found that SP600125 inhibited c-JUN activation (decreased p-c-JUN) (Figure 5A) and was markedly effective at suppressing inflammatory genes induced by HDACi treatment in a dose-dependent manner (Figure 5B). SP600125, the potent ATP competitive JNK specific inhibitor, is known to decrease expression of inflammatory mediators [37]. JNK-IN-8, which inhibits JNK1/2, suppressed c-JUN phosphorylation (p-c-JUN, Figure 5A) and silenced HDACi-induced inflammatory gene expression in a dose-dependent manner (Figure 5C). In contrast, JNK-IN-9 (a JNK2/3 specific inhibitor) and SR3576 (a JNK3 specific inhibitor) failed to block c-JUN phosphorylation (Figure 5A) or suppress HDACi-induced gene expression changes in PDAC CAFs (Figure 5D-5E), indicating that JNK1 inhibitors specifically block c-JUN activation and expression of inflammatory mediators in response to HDACi in PDAC CAFs.

We next examined the functional impact of dual HDAC and JNK inhibition in two heterocellular PDAC culture models. First, to determine the effects of JNK inhibition specifically in SAHA-treated CAFs on TC behavior, CM was generated from CAFs pre-treated with SAHA +/− SP600125. Viability of both PANC-1 and MIA PaCa-2 cells were decreased when cultured in CM from SP600125/SAHA treated CAFs as compared with SAHA controls (Figure 5F). Next, to evaluate the phenotype of TCs in the presence of CAFs and dual SAHA/JNK inhibitor treatment, the 3D TC:CAF co-culture model was employed (Figure 5G). Here, addition of SP600125 suppressed the SAHA-induced spike in CAF co-cultured PANC-1 TC proliferation on Days 1 and 2 (Figure 1D and 5G). Taken together, these data show that JNK inhibition can overcome the SAHA-induced tumor-supportive inflammatory response from CAFs, thus potentially isolating the beneficial effects on TCs and overall efficacy of HDACi's in this notoriously difficult to treat cancer and other solid tumors with a CAF laden stromal compartment.

## DISCUSSION

HDAC inhibitors effectively kill PDAC TCs in culture and pre-clinical *in vivo* models but have been unsuccessful in early phase clinical trials. Here we show that the poor clinical efficacy of HDACi's may be mediated partly by their effect on the PDAC stromal microenvironment.

**Figure 6:**
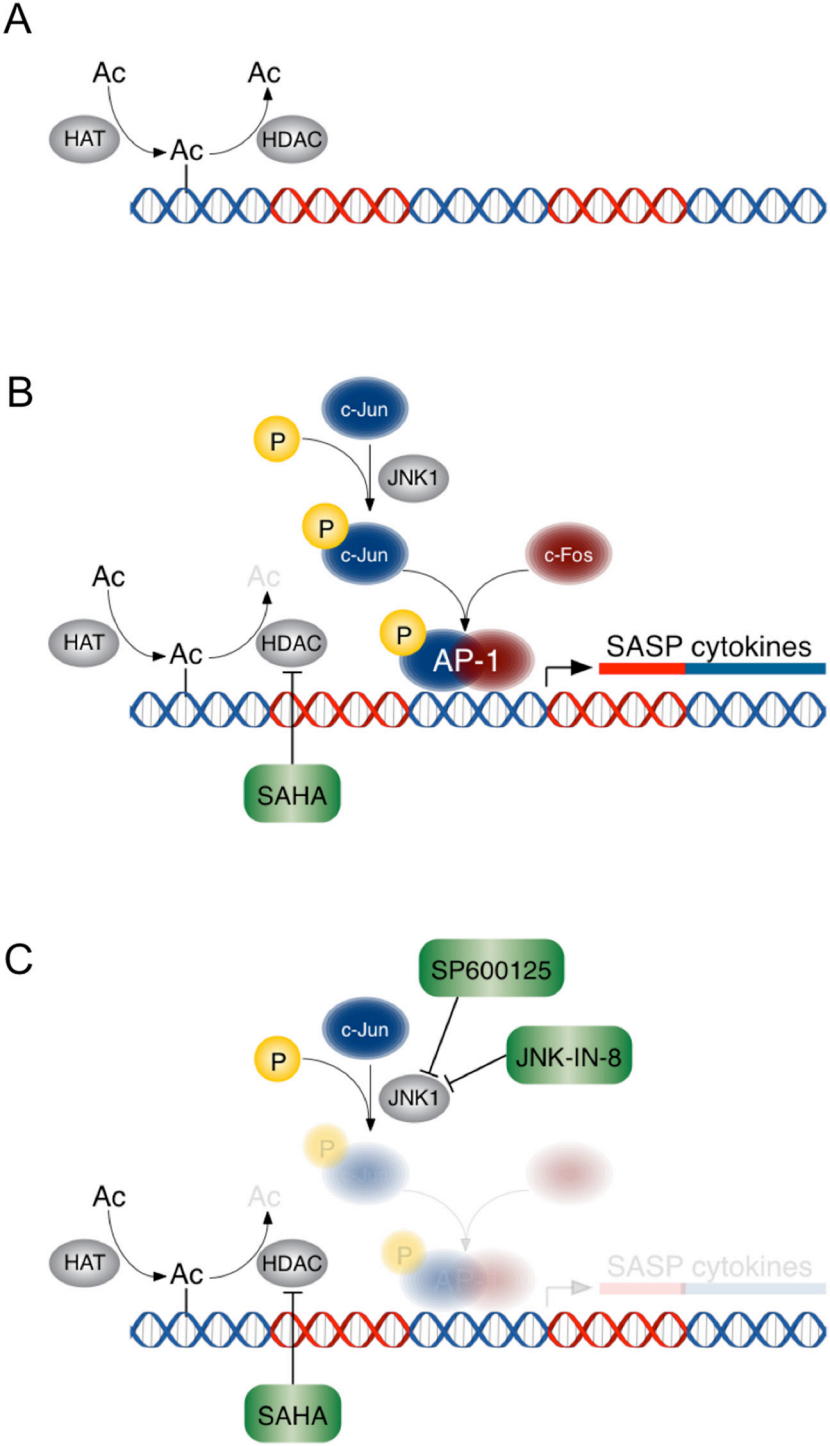
A schematic of the effects of HDACi's +/− JNK inhibition in PDAC CAFs HDAC inhibition results in hyperacetylation of histones and allows permissive gene expression of AP-1 target genes. **A**. HATs and HDACs regulate DNA acetylation. **B**. HDAC inhibition creates a permissive transcriptional landscape for SASP pro-inflammatory gene expression, which is mediated by transcription factor AP-1. **C**. JNK1 inhibition prevents pro-inflammatory gene expression despite HDAC inhibition.

As depicted in our proposed model (Figure 6), primary human PDAC CAFs treated with HDACi's develop a counter-productive and paradoxical tumor-supportive response, linked to the release of numerous pro-inflammatory SASP cytokines and chemokines. In part, regulation of these genes is dependent upon acetylation and deacetylation by HATs and HDACs, respectively. In CAFs, HDACs bind preferentially to enhancer and promoter regions upstream of these pro-inflammatory genes, in contrast to TCs where they directly regulate CDKN1A/p21 expression [24] as suggested by our gene ontology findings in PANC-1 TCs which identified cell cycle regulation. Inhibition of HDACs may yield a permissive landscape for the transcription factors, which include AP-1 (c-JUN/c-FOS), to drive transcription of the pro-inflammatory SASP genes in CAFs. Inhibition of JNK signaling, upstream of AP-1, attenuated the tumor supportive inflammatory response to HDACi’s.

Our findings that HDAC inhibition unleashes a pro-inflammatory cytokine release rather than cell arrest or death in CAFs is consistent with previous reports.[38]. Pazolli et al. demonstrated that chemical HDAC inhibition with trichostatin A induced the expression of pro-inflammatory osteopontin, IL-6, and IL-8 in CAFs; and an HDAC1 dominant-negative mutation that causes hyperactivation of its target genes caused a similar pro-inflammatory phenotype [39]. Additionally, it has also been shown that HDAC inhibition exaggerated the pro-inflammatory cellular response to toll-like receptor mediated ligation [40, 41], which is suggested to occur as a result of histone hyperacetylation, allowing deregulated transcription of pro-inflammatory genes.

In PDAC and other cancers characterized by a prominent desmoplastic stroma, pro-inflammatory stromal fibroblasts have been described to enhance TC survival and chemoresistance [15, 42, 43]. Our lab recently identified that this CAF phenotype can be accentuated by exposure to DNA damaging chemotherapies [16]. In the current study, we find that HDAC inhibition, which creates a permissive transcriptional landscape for pro-inflammatory gene expression, causes increased secretion of a variety of tumor supportive cytokines and chemokines from PDAC CAFs. We demonstrate that HDAC inhibition drives an inflammatory response in CAFs that is counterproductive and tumor supportive.

By identifying HDAC2 binding patterns in CAFs and querying ENCODE project data, we found that the transcription factor AP-1 may regulate the changes in gene expression enabled by HDACi’s. AP-1, which becomes active via phosphorylation of c-JUN by JNKs, is a crucial component involved in the expression of tumor-supportive cytokines and chemokines. JNKs occur in three isoforms (JNK 1, 2, or 3) [36] and have been shown to be activated in inflammatory diseases, including experimental models of pancreatitis [44, 45] and cancer [46]. We propose that specifically JNK1 inhibition may be additive or synergistic with HDACi's in PDAC, as we demonstrated that both the non-specific JNK (1, 2, and 3) inhibitor SP600125 or the JNK1/2 selective inhibitor JNK-IN-8 suppress the pro-inflammatory gene expression induced in PDAC CAFs by SAHA. We also find that these JNK inhibitors reduced the presence of phosphorylated c-JUN, thus decreasing the availability of the active AP-1 heterodimer, downregulating AP-1-dependent transcription, and arresting the production of tumor-supportive inflammation. While it has been previously suggested that JNK3 is a key isoform in the amplification, but not the initiation, of inflammation signaling [33], we found that JNK2/3 or specific JNK3 inhibition was ineffective in suppressing phosphorylation of c-JUN and did not alter HDACi-induced inflammatory gene expression in PDAC CAFs. In addition, SP600125 has been previously shown to enhance G1 arrest on PDAC TCs [34] which may also prove beneficial with HDACi's that induce p21 expression and subsequent cell cycle arrest in TCs.

JNK inhibitors are a particularly attractive therapy for PDAC. In addition to our current work which illustrates their tumor suppressive effects on CAFs treated with HDACi’s, they have also been previously shown to also exert anti-tumor effects directly on PDAC tumor cells: inhibiting TC proliferation in culture [34] and decreasing primary human tumor xenograft growth *in vivo* [35].

In conclusion, traditional therapies for the treatment of PDAC target rapidly proliferating TCs with cytotoxic agents. However, this TC centric view of cancer therapy can result in unanticipated and paradoxically deleterious tumor-supportive effects in the adjacent CAFs [16]. Recently, there has been greater recognition of the contribution of the tumor stroma to cancer progression and therapy resistance. We now provide further evidence the pan-HDACi SAHA dramatically increases the expression of tumor-supportive pro-inflammatory mediators in stromal fibroblasts. We identify the AP-1 transcription factor likely to be present at HDAC2 binding sites and in a focused chemical inhibitor screen, identify compounds that can suppress this counterproductive pro-inflammatory response. Finally, we provide experimental evidence that inflammation-suppressing JNK inhibition could potentially be a rational addition to HDACi treatment in PDAC. This work provides important insights into a strategy to improve treatment outcomes in stroma-rich solid organ malignancies with HDAC inhibitors.

## MATERIALS AND METHODS

### Cell lines

PANC-1 and MIA PaCa-2 pancreatic cancer cells were purchased from the American Type Culture Collection (ATCC). Short tandem repeat analysis was performed by ATCC. PANC-1 GFP pancreatic cancer cells were a gift from Dr. Huan Meng (UCLA, Los Angeles, CA) and MIA PaCa-2 GFP pancreatic cancer cells were a gift from Dr. David Dawson (UCLA, Los Angeles, CA). hTERT immortalized pancreatic cancer associated fibroblasts were gifts from the laboratory of Dr. Rosa Hwang (MD Anderson, Houston, TX). KPC-luc cells were a gift from Dr. Caius Radu (UCLA, Los Angeles, CA). KrasLSL.G12D/+; p53R172H/+; PdxCretg/+ (KPC) cells were a gift from Dr. Guido Eibl (UCLA, Los Angeles, CA). All cell lines were maintained in DMEM/F12 containing 10% FBS, 1% GlutaMax, and 1% penicillin/streptomycin and were routinely tested for Mycoplasma.

### Human primary pancreatic fibroblast isolation

Fresh pancreatic tissue was obtained from the Translational Pathology Core Laboratory from patients undergoing pancreatic resection at the Ronald Reagan UCLA Medical Center. All samples were obtained from consenting patients in accordance with policies and practices of the Institutional Review Board and the Office of the Human Research Protection Program at UCLA. Tumor samples underwent mechanical and enzymatic digestion and then digested tissues were cultured in DMEM/F12 (Corning) containing 10% fetal bovine serum (FBS) (Omega Scientific), 1% GlutaMax, and 1% penicillin/streptomycin (both Life Technologies). Newly establish primary fibroblast cultures were treated with 25 μg/ml Plasmocin (Invivogen) for two weeks and then maintained at 2.5 μg/ml. Because these are a finite resource, it was not feasible to use the same line in all cases. Primary pancreatic fibroblasts were used between passage numbers 3 to 6. Primary fibroblasts were characterized by wild-type KRAS status and α-smooth muscle actin positivity as previously described [47].

### Chemical inhibitors

The following chemical inhibitors were dissolved in DMSO (Sigma) (unless stated otherwise) as stock solutions and then subsequently diluted in culture media: Vorinostat/SAHA (Biotang), LBH-589 (Biotang), MS-275 (Biotang), SP600125 (LC Lab), JNK-IN-8 (Selleck Chem), JNK-IN-9 (Selleck Chem), and SR3576 (ApexBio).

### Conditioned media generation

Primary pancreatic fibroblasts were grown to 90% confluence. Fibroblasts were treated with HDACi, HDACi combined with a chemical inhibitor, or DMSO control for 24 hours. Plates were then washed and cells were incubated with phenol red free, serum-free DMEM/F12 containing 1% GlutaMax and 1% penicillin/streptomycin. After 24 hours incubation, conditioned media was harvested, 0.22 μm filtered, and then stored in aliquots at -20°C.

### *In vitro* cell viability

Cells were seeded in 96-well plates with doses of HDACi's diluted in media in a total volume of 100 μl per well. At indicated time points, 10 μl of 5 mg/ml MTT (Life Technologies) in PBS was added to each well and incubated for 4 hours at 37°C and 5% CO_2_. Cells were then lysed with 100 μl 10% SDS (Sigma), 0.01M HCl (Sigma) and incubated overnight at 37°C and 5% CO_2_. Absorbance readings were measured at 560 nm wavelength using a Modulus II Microplate Multimode Reader (Turner BioSystems). Alternatively, cell viability was measured using the CellTiter-Glo Luminescent Cell Viability Assay (Promega) according to the manufacturer's recommendations. Experiments were performed in triplicate wells and repeated at least twice.

### *In vitro* tumor cell-fibroblast 3D co-culture

40 μl of Matrigel (Corning) was plated in black walled, flat bottom 96 well plates (Corning) and allowed to polymerize for 30 minutes at 37°C and 5% CO_2_. 5 × 10^3^ PANC-1 GFP cells and 1 × 10^4^ primary pancreatic fibroblasts were seeded together with HDACi's with or without other chemical inhibitors in 100 μl DMEM/F12 containing 10% FBS, 1% GlutaMax, and 1% penicillin/streptomycin and 2% Matrigel. In experiments with PANC-1 cells were cultured alone, 5 × 10^3^ PANC-1 GFP were used. Fluorescence readings were measured using a blue optical kit (Ex 490 nm/Em 510-570 nm) on a Modulus II Microplate Multimode Reader. Images were taken using a CX41 Inverted Microscope with a DP26 Digital Camera (Olympus). Experiments were performed in triplicate wells and repeated at least twice.

### *In vitro* tumor cell proliferation

PANC-1 GFP and MIA PaCa-2 GFP cells were plated in black walled, flat bottom 96 well plates at 3 × 10^3^ cells per well in fibroblast conditioned media containing 1% FBS. Fluorescence readings were measured daily using a blue optical kit (Ex 490 nm/Em 510-570 nm) on a Modulus II Microplate Multimode Reader. Experiments were performed in triplicate wells and repeated at least twice.

### *In vivo* xenograft tumor growth

All mouse experiments were conducted in accordance with protocols approved by the Institutional Animal Care and Use Committee at the University of California, Los Angeles. 5 × 10^5^ PDAC tumor cells were resuspended in 1:1 diluted Matrigel in DMEM/F12 1% GlutaMax and 1% penicillin/streptomycin and then injected in 100 μl volumes in the subcutaneous flank five 6-8 week old sex-matched NOD/SCID/IL2γ mice. Alternatively, in our orthotopic KrasLSL.G12D/+; p53R172H/+; PdxCretg/+ (KPC) PDAC mouse model, KPC tumor cells in 40 μl suspensions were implanted in the pancreata of 6-8 week old sex-matched C57BL/6 mice. For conditioned media supplementation experiments, beginning at day 7, tumors were supplemented thrice weekly with 50 μl peri-tumoral injections of conditioned media. For SAHA treatment experiments, mice were treated 5 times weekly with 40 mg/kg SAHA in PBS by oral gavage. Subcutaneous tumors were measured weekly by dial caliper or *in vivo* bioluminescence and mice were sacrificed when tumor diameters approached 15 mm.

### *In vitro* tumor cell-fibroblast invasion assay

MIA PaCa-2 and primary pancreatic fibroblasts were each seeded at 5 × 10^4^ cells per Matrigel-coated 8 μm pore PET membrane 24-well cell culture inserts (Corning) in conditioned media supplemented with 0.1% FBS. 10% FBS conditioned media was placed in the bottom wells and cells were allowed to invade for 24 hours. After completion of invasion, cells on the lower surface of the membrane were fixed and stained with hematoxylin. Images were taken using a CX41 Inverted Microscope with a DP26 Digital Camera. Invaded tumor cells were quantified in five 20X microscope visual fields based on distinct differences in morphology between primary pancreatic fibroblasts and MIA PaCa-2 cells. Experiments were performed in triplicate wells and repeated at least twice.

### Chemiluminescent cytokine array

Primary pancreatic fibroblasts were treated for 24 hours with HDACi or with DMSO alone and media was subsequently harvested. Media was assayed using the Human Cytokine Array Panel A (R&D Systems), according to manufacturer's instructions. Membranes were developed using standard chemiluminescent techniques and images were captured using the Odyssey Fc Imaging System (LI-COR). Pixel density was calculated using Image Studio software (LI-COR).

### Gene expression by quantitative real-time PCR

Total RNA was isolated from cells using the Quick-RNA MiniPrep kit (Zymo). Reverse transcription was performed using the High Capacity cDNA Reverse Transcription kit (Life Technologies). Quantitative PCR was performed using EvaGreen qPCR Master Mix (Lamda Biotech). RNA expression values were normalized to 36B4 control gene and then calculated as relative expression to control. Primers used are reported in Supplmentary Table 1.

### ChIP-Seq

Chromatin Immunoprecipitation (ChIP) was performed as described previously [48] using αHDAC2 antibody from Bethyl laboratories (Cat#A300-705/Lot#1). Sequencing libraries were constructed from 0.2ng of input and immunoprecipitated DNA using the KAPA DNA Library Preparation Kit (KapaBiosystems). Sequencing on an Illumina HiSeq 2000 sequencer yielded between 13 and 30 million reads. Analysis of sequence data was as previously described [49], except that the genome was tiled into 50 bp windows.

### Western blots

Western blots were performed using the LI-COR Odyssey infrared blot system, according to manufacturer's instructions. Blots were performed on acid-extracted histones or whole cell lysates. The following antibodies were used: p-c-JUN (Ser63) Lot #6 (Cell Signaling), GAPDH Lot #PJ20538 (ThermoFisher), acetyl-Histone-H3 Lot #2370129 (Millipore), acetyl-Histone-H4 Lot #2302181 (Millepore), H3K18ac Lot #30508004 (Active Motif), H3K27ac Lot #GR167613-1 (Abcam). LI-COR IRDye 700 and 800 nm channel secondary antibodies were used. Western blots were imaged using the Odyssey Fc Imaging System in 700 and 800 nm channels and densitometry analysis was performed using the LI-COR Image Studio software.

### Statistical analyses

Data are presented as the mean ± standard error of the mean unless otherwise stated. Statistical significance was calculated via the Student's t-test. Values of ≤0.05 were considered statistically significant. Statistical analyses were performed using Prism 6 Software (GraphPad). Transcription binding significance of the ENCODE ChIP-Seq Significance Tool results are calculated by a hypergeometric test with a multiple hypothesis correction [31].

### Abbreviations

HDAC - histone deacetylase, HDACi – histone deacetylase inhibitor, PDAC – pancreatic ductal adenocarcinoma, CAF – cancer-associated fibroblast, HAT – histone acetyltransferase, SAHA - suberanilohydroxamic acid, ChIP - chromatin immunoprecipitation, KPC - KrasLSL.G12D/+; p53R172H/+; PdxCretg/+, TC – tumor cell, CM – conditioned media, JNK - janus N-terminal kinase.

## SUPPLEMENTARY MATERIALS FIGURES AND TABLES


