# Supplementary material for: Histone deacetylase inhibitors provoke a tumor supportive phenotype in pancreatic cancer associated fibroblasts
Source: Oncotarget. 2016 Nov 24;8(12):19074–88. doi: 10.18632/oncotarget.13572 (PMC5386671; doi:10.18632/oncotarget.13572)
Supplement: Supplementary file 1 [file oncotarget-08-19074-s001.pdf]

Histone deacetylase inhibitors provoke a tumor supportive phenotype in pancreatic cancer associated fibroblasts

SUPPPLYMENTARY FIGURES AND TABLE

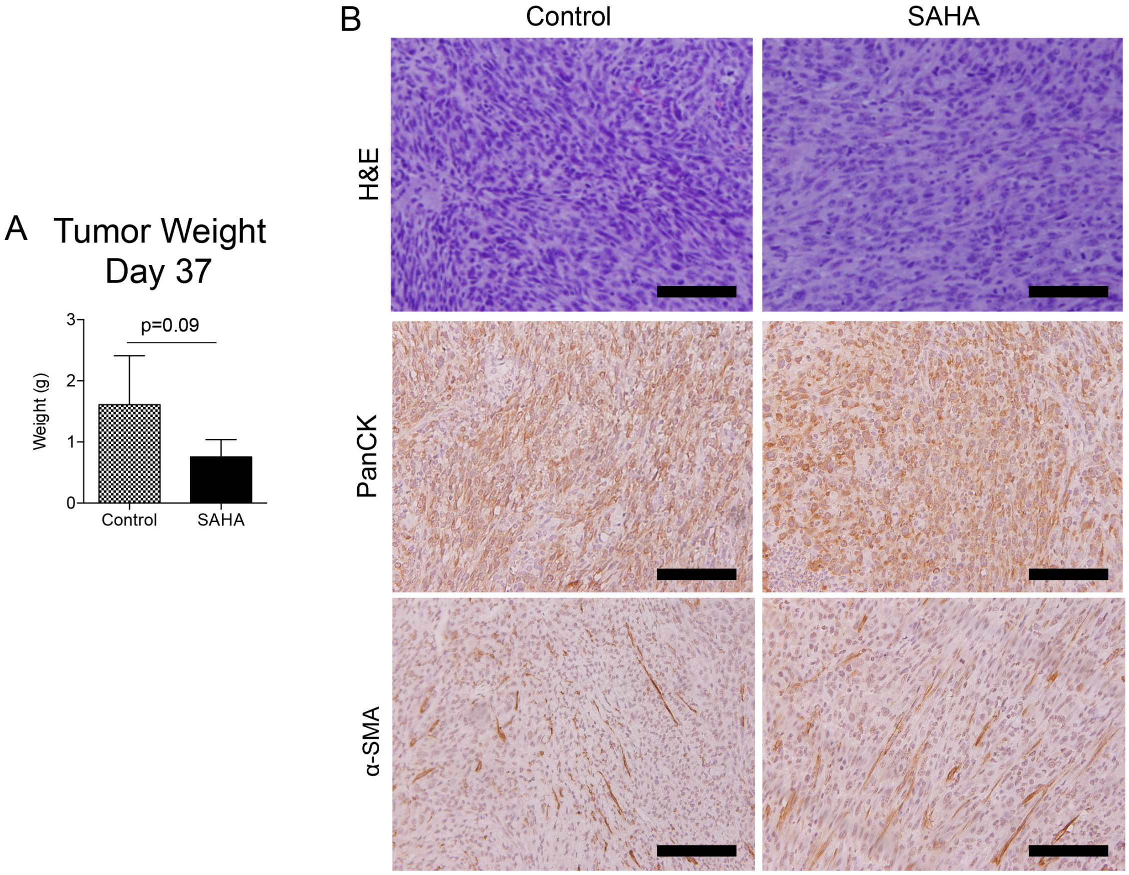

Supplementary Figure 1: KPC-luc cells were implanted in the pancreata of C57BL/6 mice and mice were treated with oral gavage of SAHA 5 times weekly beginning on Day 7. A. Mice were sacrificed on Day 37 and tumor weights were measured. **B.** H&E and IHC for PanCK and α-SMA stains were performed on control and SAHA treated tumors (explanted on Day 37) (20X; scale: 100 μm).

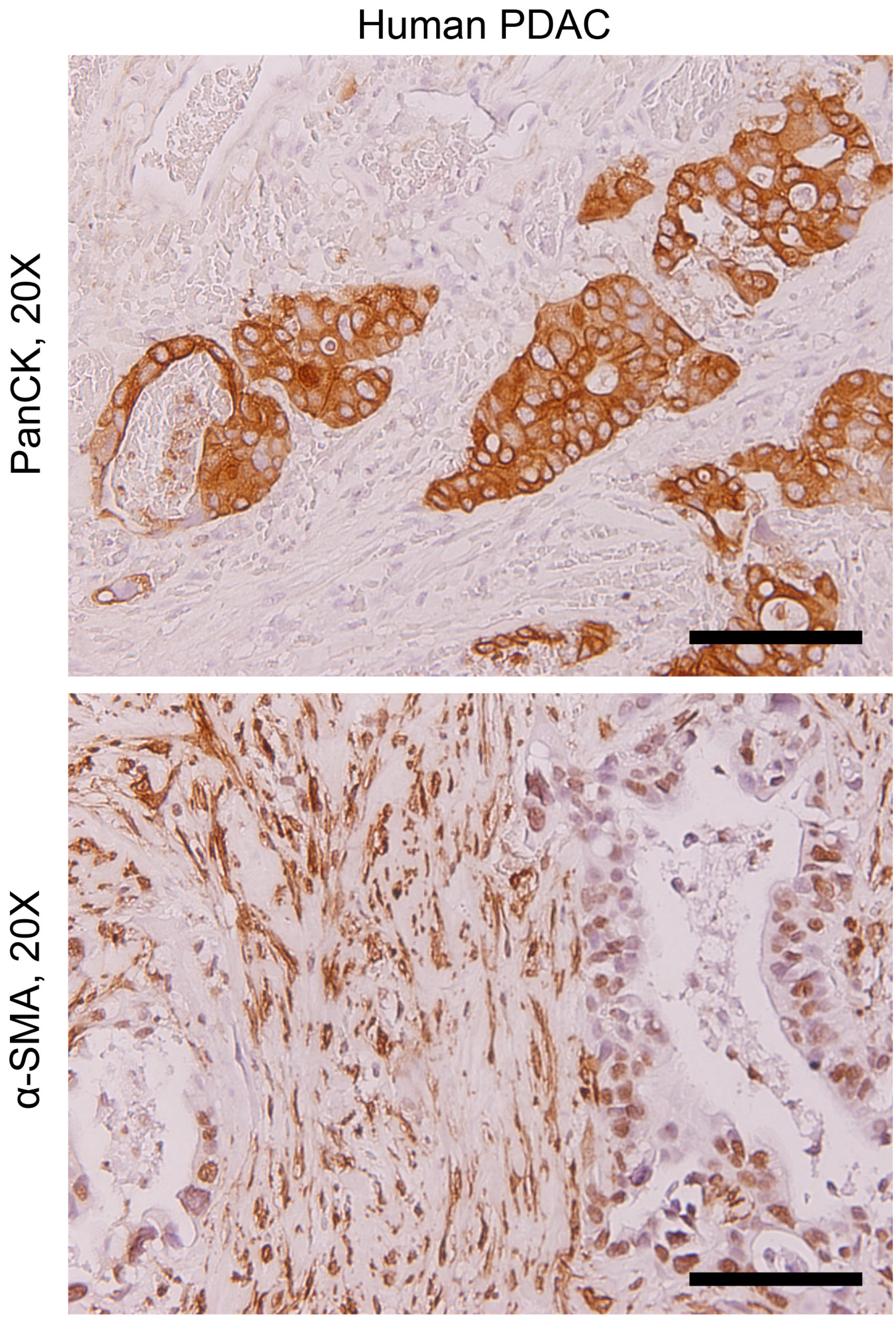

Supplementary Figure 2: Human PDAC tissue immunohistochemistry. Human PDAC tissues were stained for PanCK and α-SMA (20X; scale: 100 μm).

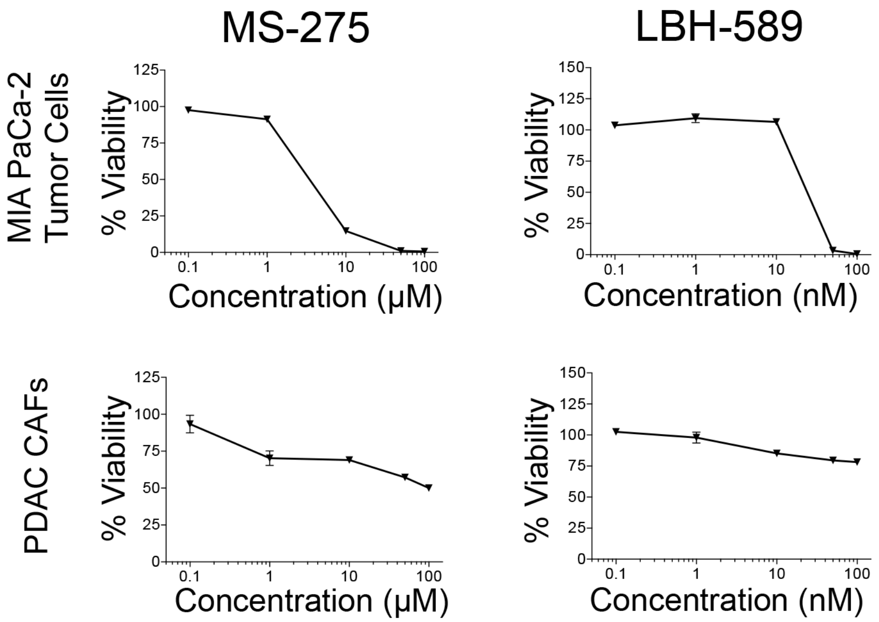

Supplementary Figure 3: MS-275 and LBH589 decrease MIA PaCa-2 cell viability but not primary PDAC CAFs. MIA PaCa-2 (above) or primary PDAC CAFs (below) were treated with either MS-275 (left) or LBH589 (right) and MTT assays performed at 96h.

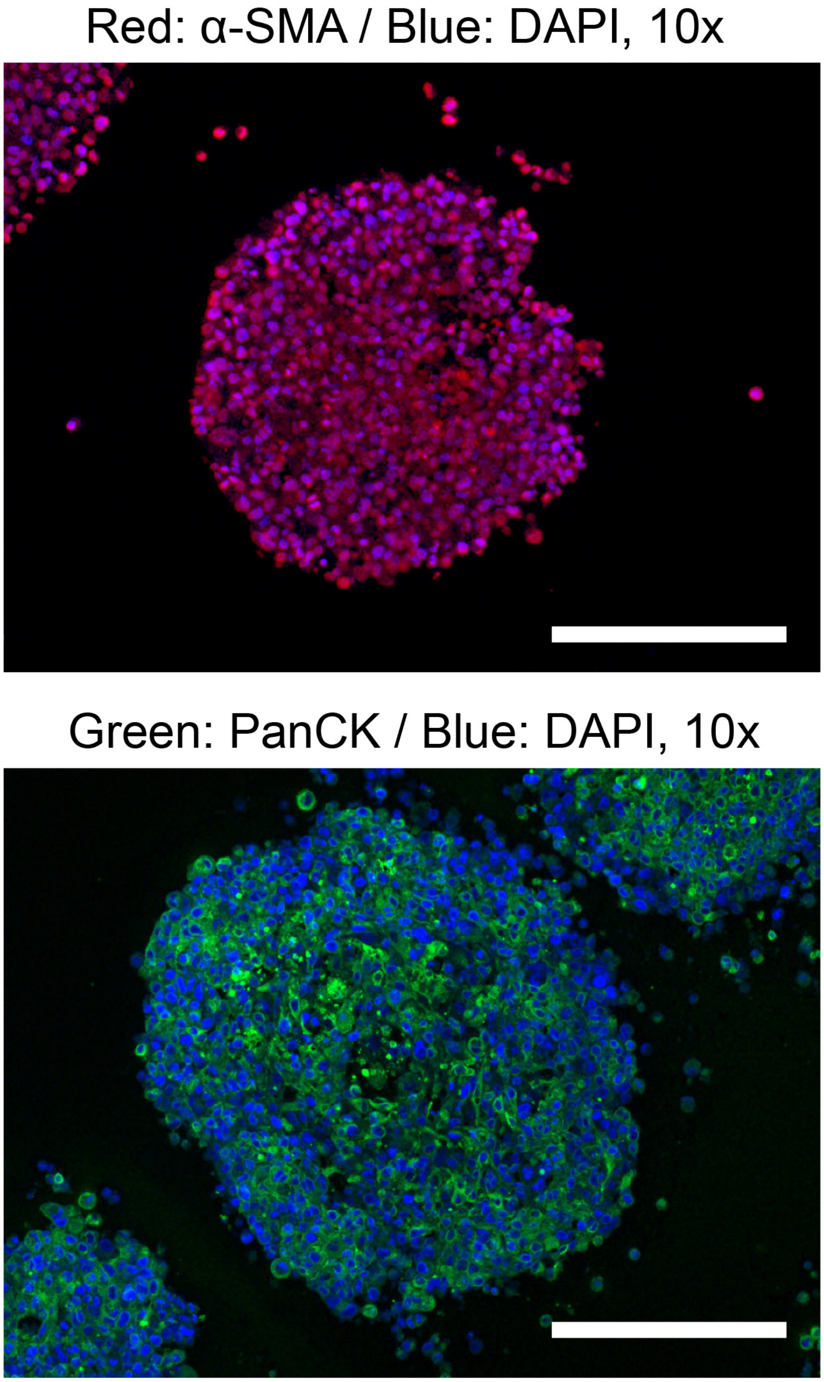

Supplementary Figure 4: 3D TC:CAF co-culture contain evenly distributed PANC-1 TC and PDAC CAFs throughout cultures. Immunofluorescence was performed of 3D cultures that were embedded and sectioned and stained with α-SMA (red) for fibroblasts and PanCK (green) for cancer cells. DAPI stains cell nuclei (10X; scale: 200 µm).

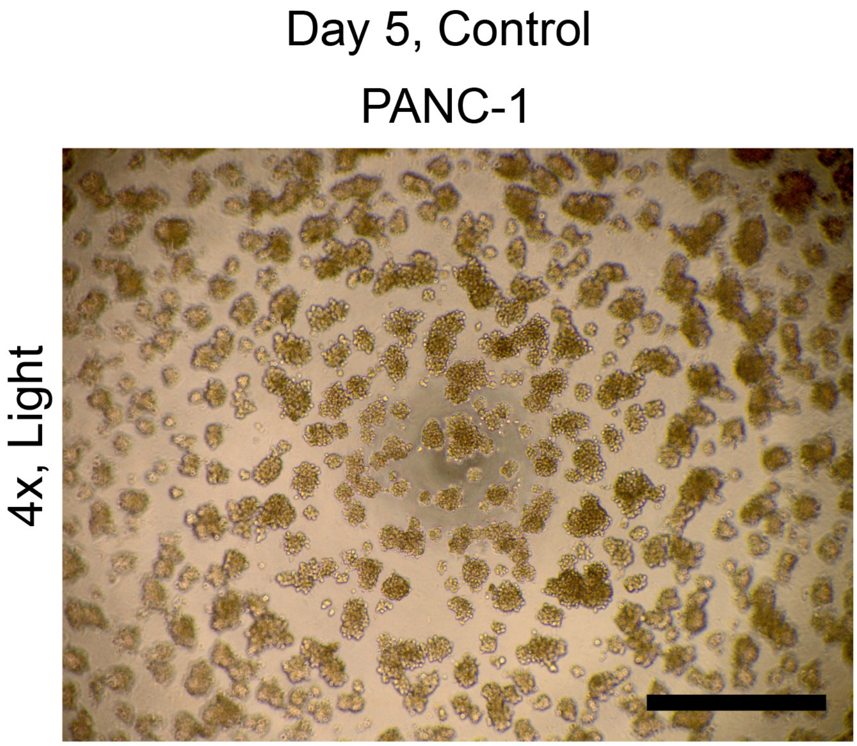

Supplementary Figure 5: Untreated PANC-1 cells cultured in the absence of CAFs on Matrigel after 5 days. Light inverted microscopy image of PANC-1 cells cultured on Matrigel (4X; scale: 400 µm).

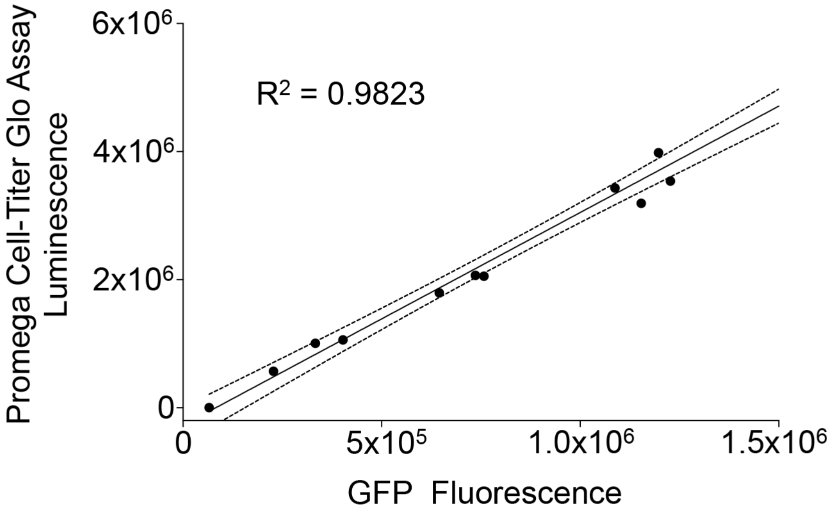

Supplementary Figure 6: Validation of fluorescence measurements of GFP expressing cells using the Promega Cell-Titer Glo luminescence assay. Promega Cell-Titer Glo cell viability luminescence assay was performed in serial numbers of PANC-1-GFP cells and the relationship between luminescence and fluorescence was assessed with a linear regression model ($R^2 = 0.9823$).

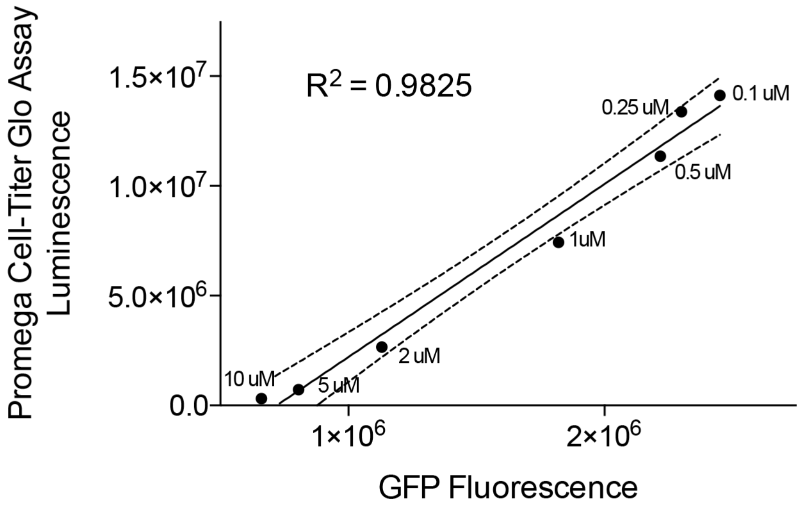

Supplementary Figure 7: Validation of fluorescence measurements of GFP expressing cells treated with SAHA using the Promega Cell-Titer Glo luminescence assay. Promega Cell-Titer Glo cell viability luminescence assay was performed in PANC-1-GFP cells treated with various doses of SAHA. At the various doses, we still observed a linear relationship between viability (luminescence) and GFP expression using a linear regression model ($R^2 = 0.9825$).

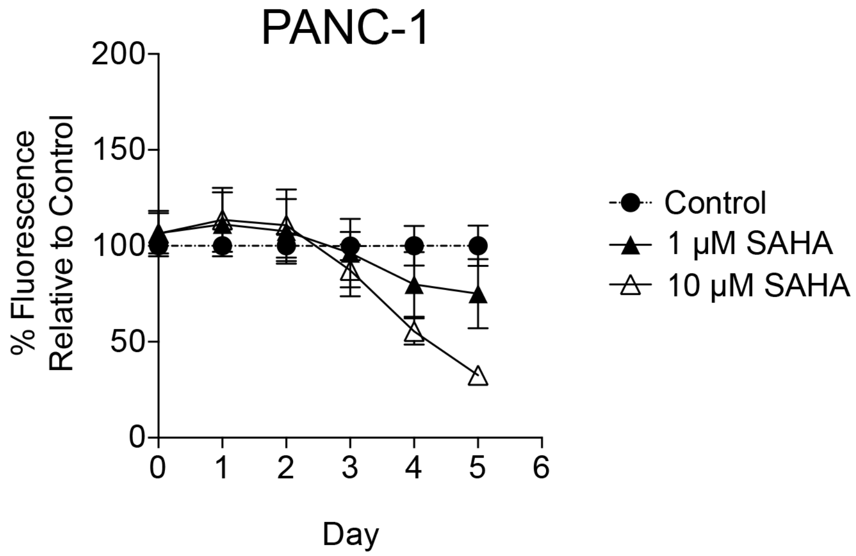

Supplementary Figure 8: Fluorescence measurements of PANC-1-GFP cells in 3D mono-culture +/- SAHA for 5 days.

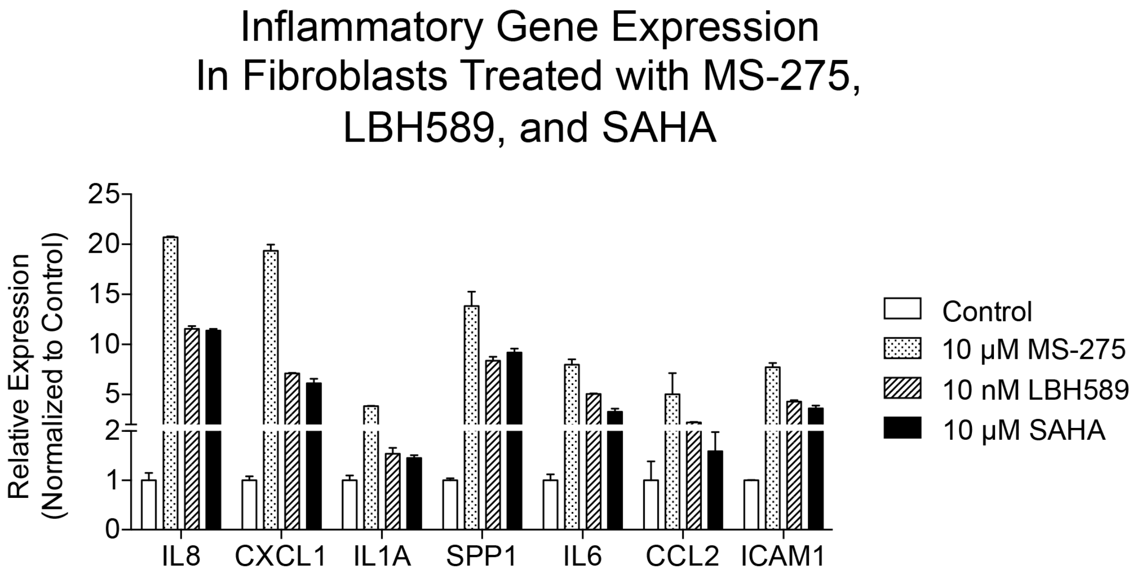

Supplementary Figure 9: Pro-inflammatory gene expression of PDAC CAFs treated with MS-275, LBH589 and SAHA. Primary PDAC CAFs were treated for 24h with 10 μM MS-275, 10 nM LBH589 or 10 μM SAHA and expression was determined of a panel of pro-inflammatory genes by qRT-PCR.

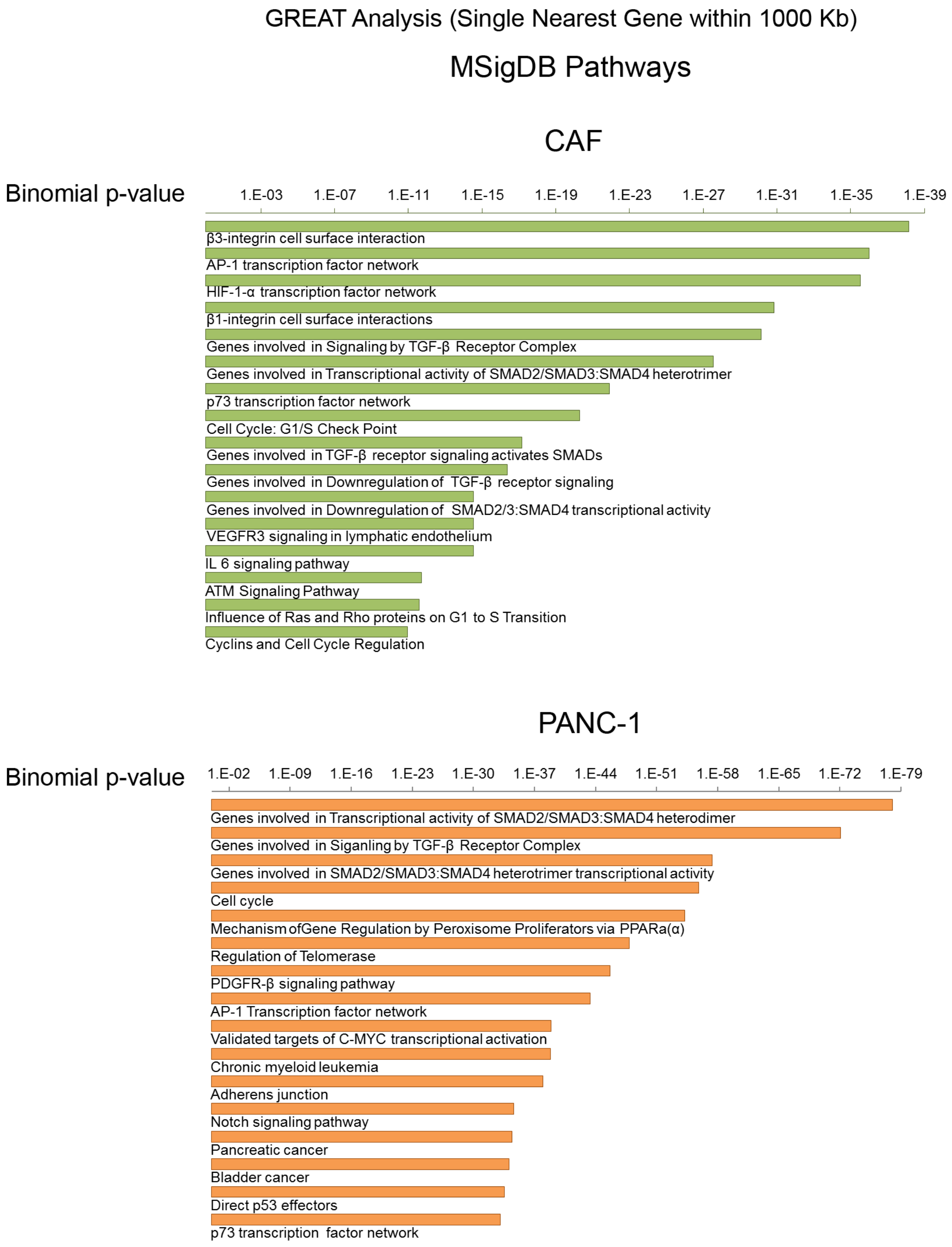

Supplementary Figure 10: Using the GREAT: Genomic Regions Enrichment of Annotations Tool (http://bejerano.stanford.edu/great/public/html/index.php), we identified biologic functions associated with the sites of HDAC2 binding in CAFs and in PANC-1 TCs with analysis selecting the single nearest gene within 1000 Kb from HDAC2 binding sites.

SeqPos Transcription factor motif search

CAF peaks

factor	DNA binding domain	hits	cutoff	zscore	-10*log(pval)	mean_position
JUNB	Leucine Zipper Family	1539	5.953	-30.217	690.776	-0.172
Jund	Leucine Zipper Family	1403	6.851	-21.404	690.776	-0.131
NFE2L2	Leucine Zipper Family	3276	2.593	-17.963	690.776	-0.073
Cebpb	Leucine Zipper Family	808	7.481	-12.581	690.776	-0.109
ATF4	Leucine Zipper Family	1636	4.713	-10.432	576.886	-0.065
ATF4	Leucine Zipper Family	4980	-0.633	-10.287	561.661	-0.037
NFIL3	Leucine Zipper Family	1076	6.641	-9.821	514.439	-0.076
TEAD3	Homeodomain Family	1614	5.996	-9.044	440.328	-0.058
Rest	BetaBetaAlpha-zinc finger Family	46	13.733	-8.088	357.285	-0.323
MYC	Helix-Loop-Helix Family	2186	5.056	-7.699	326.102	-0.044

PANC-1 peaks

factor	DNA binding domain	hits	cutoff	zscore	-10*log(pval)	mean_position
JUNB	Leucine Zipper Family	687	7.413	-11.566	690.776	-0.11
HEY1	Helix-Loop-Helix Family	4249	2.539	-11.334	675.896	-0.043
E2F6	Transcription Factor Family	3619	3.81	-10.899	627.11	-0.045
Ctcf	BetaBetaAlpha-zinc finger Family	1192	5.019	-10.063	538.739	-0.074
MYC	Helix-Loop-Helix Family	3335	4.157	-9.92	524.236	-0.043
Zscan10	BetaBetaAlpha-zinc finger Family	4829	-0.923	-8.28	373.247	-0.031
HES5	Helix-Loop-Helix Family	3772	0.034	-7.861	338.963	-0.034
ZFP42	BetaBetaAlpha-zinc finger Family	390	8.347	-7.671	323.961	-0.105
Tbx3	Transcription Factor T-Domain	4070	3.238	-7.499	310.655	-0.032
ERG	Ets Domain Family	2981	4.448	-7.363	300.412	-0.036

Supplementary Figure 11: Using the Cistrome SeqPos motif analysis, we identified transcription factors whose binding motifs are enriched within 600 bp of HDAC2 binding in CAFs and PANC-1 TCs.

Supplementary Table 1: A list of primers for qRT-PCR gene expression analysis

Gene symbol	Primer	Primer sequence (5' to 3')
IL8	Forward	ACTGAGAGTGATTGAGAGTGGAC
	Reverse	AACCCTCTGCACCCAGTTTTC
CXCL1	Forward	AGGGAATTCACCCCAAGAAC
	Reverse	ACTATGGGGGATGCAGGATT
IL1A	Forward	CGCCAATGACTCAGAGGAAGA
	Reverse	AGGGCGTCATTCAGGATGAA
SPP1	Forward	GCCGAGGTGATAGTGTGGTT
	Reverse	TGAGGTGATGTCCTCGTCTG
IL6	Forward	AAAGAGGCACTGGCAGAAAA
	Reverse	AGCTCTGGCTTGTTCCTCAC
CCL2	Forward	GCCTCCAGCATGAAAGTCTC
	Reverse	CACTTGCTGCTGGTGATTCT
ICAM1	Forward	GCTGACGTGTGCAGTAATACTGG
	Reverse	TTCTGAGACCTCTGGCTTCGT
36B4	Forward	GTGCTGATGGGCAAGAAC
	Reverse	AGGTCCTCCTTGGTGAAC
